# Regulation of shrimp prophenoloxidase activating system by lva-miR-4850 during bacterial infection

**DOI:** 10.1038/s41598-021-82881-2

**Published:** 2021-02-15

**Authors:** Pakpoom Boonchuen, Phattarunda Jaree, Kulwadee Somboonviwat, Kunlaya Somboonwiwat

**Affiliations:** 1 Center of Excellence for Molecular Biology and Genomics of Shrimp, Department of Biochemistry, Faculty of Science, Chulalongkorn University, Bangkok, Thailand; 2 Center of Applied Shrimp Research and Innovation, Institute of Molecular Biosciences, Mahidol University, Salaya, Nakhon Pathom Thailand; 3 Faculty of Engineering at Sriracha, Kasetsart University Sriracha Campus, Sriracha, Chonburi Thailand; 4 Omics Science and Bioinformatics Center, Faculty of Science, Chulalongkorn University, Bangkok, Thailand

**Keywords:** RNAi, Bacterial infection

## Abstract

MicroRNAs (miRNAs) suppress gene expression and regulate biological processes. Following small RNA sequencing, shrimp hemocytes miRNAs differentially expressed in response to acute hepatopancreatic necrosis disease (AHPND) caused by *Vibrio parahaemolyticus* (VP_AHPND_) were discovered and some were confirmed by qRT-PCR. VP_AHPND_-responsive miRNAs were predicted to target several genes in various immune pathways. Among them, lva-miR-4850 is of interest because its predicted target mRNAs are two important genes of the proPO system; *proPO2* (*PO2*) and *proPO activating factor 2* (*PPAF2*). The expression of lva-miR-4850 was significantly decreased after VP_AHPND_ infection, whereas those of the target mRNAs, *PO2* and *PPAF2*, and PO activity were significantly upregulated. Introducing the lva-miR-4850 mimic into VP_AHPND_-infected shrimps caused a reduction in the *PO2* and *PPAF2* transcript levels and the PO activity, but significantly increased the number of bacteria in the VP_AHPND_ targeted tissues. This result inferred that lva-miR-4850 plays a crucial role in regulating the proPO system via suppressing expression of *PPAF2* and *PO2*. To fight against VP_AHPND_ infection, shrimp downregulated lva-miR-4850 expression resulted in proPO activation.

## Introduction

Acute hepatopancreatic necrosis disease (AHPND) caused by *Vibrio parahaemolyticus* VP_AHPND_ carrying a toxin-encoded plasmid is responsible for severe losses in the shrimp industry^1^. There is no current effective approach to prevent VP_AHPND_ infection. The AHPND’s characteristic symptoms were reported as a pale and atrophied hepatopancreas (HP) together with an empty stomach and midgut^2^. Histological examination of the HP, the AHPND target tissue, further showed that AHPND causes sloughing of the HP tubule epithelial cells into the HP tubule lumens^3^. A metabolic utilization analysis of VP_AHPND_ indicated that VP_AHPND_ could utilize 23 nutrient sources more efficiently than the other non-pathogenic VP strains, as ascertained by their significantly greater growth^4^.

MicroRNAs (miRNAs) are small non-coding RNA molecules that function in RNA silencing and post-transcriptional regulation of gene expression^5^. During biogenesis, miRNAs of 18–24 double-stranded oligonucleotides are generated. Following unwinding, the single-stranded mature miRNA, or so-called guide strand, is integrated into an RNA-induced silencing complex (RISC) resulting in the active miRNA-RISC (miRISC) complex. The miRISC interacts with mRNA targets via complementary base pairing between the “seed” region of the miRNA (typically nucleotides 2–8) and the target sequence on the mRNA inducing mRNA degradation and/or translational repression^6^.

In animals, miRNA expression studies in response to various bacterial infections have revealed common miRNAs as key players in the host innate immune response^7^. For example, miR-146 along with miR-155, were found to be coordinately unregulated in immune cells in response to various bacterial pathogens, including *Salmonella enteric*^8,9^, *Mycobacterium* species^10–14^, and *Francisella tularensis*^15^.

Expression of miR-155 in mice is induced by both bacterial and viral compounds through Toll-like receptors (TLRs) sensing bacterial and viral pathogens, and also by tumor necrosis factor-α and the antiviral interferons^16,17^. In shrimps, various immune functions of host miRNAs have been characterized^18^. Previously, 23,365 known miRNAs and 481 novel miRNAs from *Marsupenaeus japonicus* induced upon white spot syndrome virus (WSSV) infection were found to be involved in the regulation of several immune-related gene targets, such as *cathepsin*, *c-type lectin*, *hemocyanin*, and *ubiquitin protein ligase*^19^. In addition, bacterial-responsive miRNAs have been identified in the shrimp *M. japonicus*, and some of them have been predicted to play a role in apoptosis^20^. However, the information of miRNAs in the antibacterial immune response against VP_AHPND_ infection and their target genes is still limited.

To understand the interaction between shrimp miRNAs and the target genes that affect their health upon VP_AHPND_ infection, it is necessary to identify the set of VP_AHPND_-responsive miRNAs that participate as key regulators. An understanding of the regulation of gene networks could allow the potential therapeutic use of such key regulator miRNAs to combat infection^21^. In the present study, VP_AHPND_-responsive miRNAs of *Penaeus vannamei* hemocytes were discovered using small RNA sequencing (sRNA-Seq). Differentially expressed miRNAs were further verified by qRT-PCR. The immune-related miRNA, lva-miR-4850, was further characterization for its function in the shrimp antibacterial immune response, particularly on the prophenoloxidase (proPO) activating system.

## Results

### Identification of miRNAs from hemocytes of VP_AHPND_-challenged shrimps

**Figure 1:**
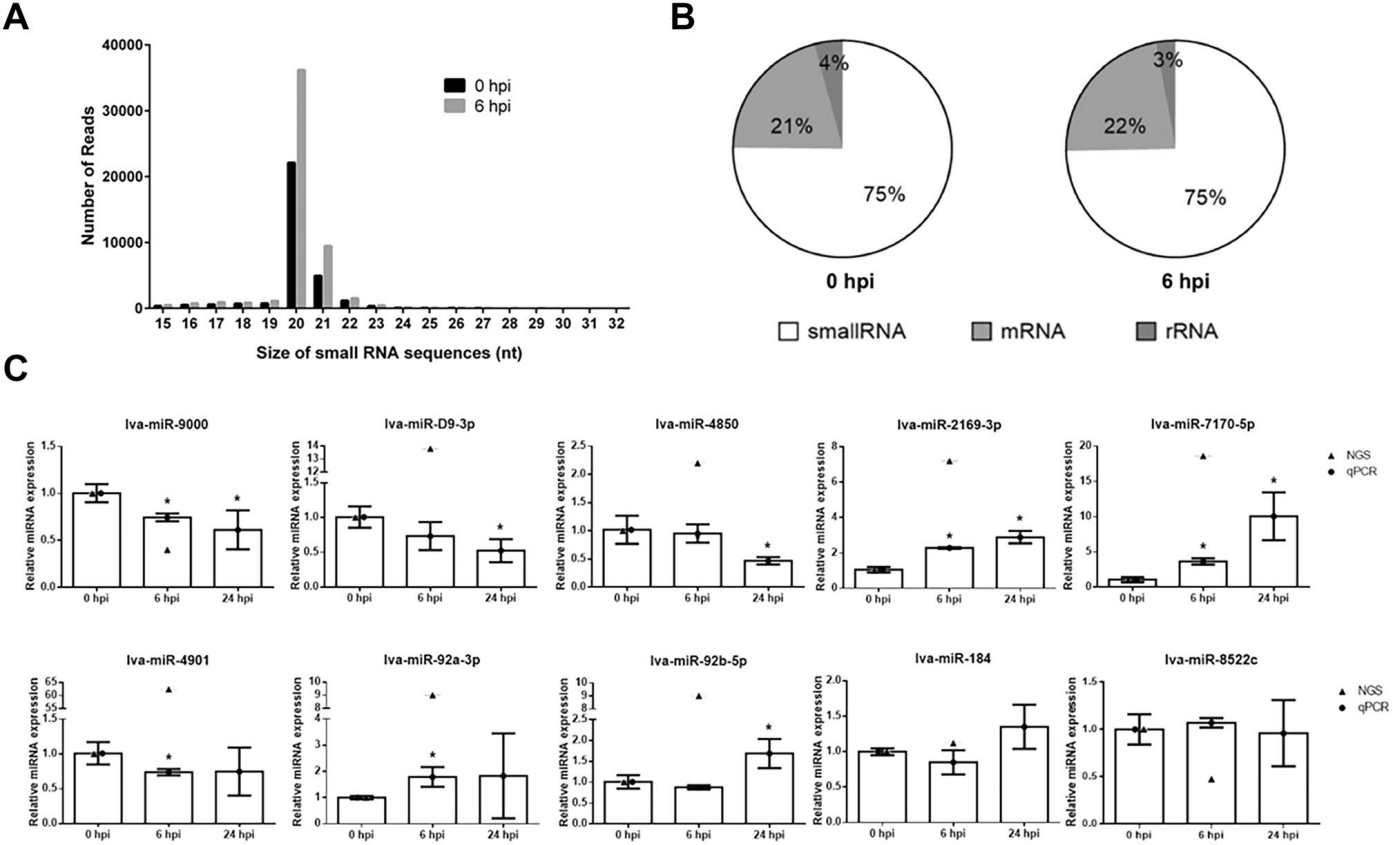
Transcriptomic profiles of miRNAs from *P. vannamei* hemocytes in response to VP_AHPND_ infection. (**A**) Length distribution and abundance of sRNAs from hemocytes of VP_AHPND_-challenged *P. vannamei* at 0 and 6 hpi. (**B**) Composition of RNAs in each sRNA-Seq library. (**C**) Total RNA from VP_AHPND_-infected *P. vannamei* hemocytes was used as a template for specific stem-loop first-strand cDNA synthesis. The relative expression levels of 10 miRNAs were determined by qRT-PCR at 0, 6, and 24 hpi and normalized against U6 as the internal reference. Data are shown as the mean ± 1 SD, derived from three independent repeats. Asterisks indicate significant differences at *P* < 0.05 (DMRT).

The global analysis of miRNA expression in shrimps challenged with VP_AHPND_ was performed by sequencing small RNA (sRNA) libraries derived from hemocyte samples collected at 0 and 6 h post-infection (hpi) with VP_AHPND_. High-throughput sequencing generated a total of 2,184,366 raw reads, comprised of 931,638 and 1,252,728 raw reads in the 0 and 6 hpi sRNA libraries, respectively (Table S1). A total of 1,921,212 reads from both libraries were high quality sequences that passed the initial quality filters. Among them, the majority of non-redundant sequences were 20–22 nucleotides (nt) long (Fig. 1A). Searching against the NCBI nucleotide database demonstrated that, on average, 25% of the sequences were likely contaminating RNAs (Fig. 1B). Following the removal of these contaminating mRNAs, rRNA, and tRNA homologs, the final sequence count was 62,567 sequences that were mapped to miRBase 22.1. The percentage of matched mature miRNA sequences in the 0 and 6 hpi libraries was 94.98% and 96.00%, respectively. A total of 620 miRNA homologs were identified from both libraries. Of those, only 20 miRNA homologs were identified as putative differentially expressed miRNAs (DEMs) upon VP_AHPND_ infection (Table 1).

**Table 1:** Nucleotide sequences of differentially expressed miRNA homologs identified in VP_AHPND_-infected *P. vannamei* hemocytes.

miRNA name	Sequence (5′–3′)	log_2_(Fold change)	*P-*value
lva-miR-2044-3p	GAAAAAUUAUCUUGAUAAAGC	− 4.2	5.31E−04
lva-miR-71	UCUCACUACCUUGUCUUUCACG	− 2.54	3.83E−02
lva-miR-745-3p	GAGCUGCCCAAUGAAAGGCUG	− 2.1	5.59E−07
lva-miR-965	UAAGCGUAUGGCUUUUCCCCU	− 1.58	1.47E−16
lva-miR-9000	AAGCCCCAGUGGCGCAAUCG	− 1.32	1.66E−07
lva-miR-100-5p	AACCCGUAGAUCCGAACUUGUG	− 1.16	4.11E−25
lva-miR-7427-5p	AGAAACGCGGCACAGAAU	− 1.15	2.55E−25
lva-miR-305	AUUGUACUUCAUCAGGUGCUCG	− 1.12	4.32E−26
lva-miR-8552c	GGCCGUGAUCGUAUAGUG	− 1.08	7.95E−27
lva-miR-184	UGGACGGAGAACUGAUAAGGG	1.08	2.48E−03
lva-miR-4850	AUAACAUGACUGAAAACAUUU	1.14	1.45E−28
lva-miR7695-3p	CGAUUGUGCCACGGAGGCAU	1.14	3.20E−07
lva-miR-2238j-5p	UCGUCAGCUCCAUCCGCAAGG	1.26	1.66E−07
lva-miR-5395	GGCGAGCGAAAUUGGACUAGC	1.26	1.66E−07
lva-miR-2169-3p	AUUUAAAGUGGUACGCGAGCUGG	2.85	9.49E−09
lva-miR-92a-3p	UCGUCUCGUGUCUCGGCCUUAG	3.17	5.72E−09
lva-miR-92b-5p	GGACGAGAAGCGGUGCUU	3.17	5.72E−09
lva-miR-D9-3p	UUUCCAGAAUGUUCCACU	3.79	1.76E−10
lva-miR-7170-5p	AACUGGAGGACCGAACCGACU	4.22	1.76E−10
lva-miR-4901	UAACUUAUUUUUGGACAAAC	5.96	4.05E−15

### Analysis of miRNA expression upon VP_AHPND_ infection using stem-loop qRT-PCR

In order to confirm the presence and expression of the identified DEMs, the expression level of 10 selected VP_AHPND_-responsive miRNAs (lva-miR-9000, lva-miR-8522c, lva-miR-2169-3p, lva-miR-4850, lva-miR-92b-5p, lva-miR-D9-3p, lva-miR-184, lva-miR-4901, lva-miR-7170-5p, and lva-miR-92a-3p) were evaluated using stem-loop qRT-PCR. The relative expression levels of these miRNAs were determined in VP_AHPND_-infected *P. vannamei* hemocytes at 0, 6, and 24 hpi, using U6 as the internal reference (Fig. 1C). The results indicated that 8 out of 10 miRNAs were significantly differentially expressed in shrimp hemocytes following VP_AHPND_ challenge. Unexpectedly, the expression levels of lva-miR-8522c and lva-miR-184 remained unchanged at 6 and 24 hpi. Similar to the RNA-Seq result, the expression of lva-miR-9000 at 6 and 24 hpi was down-regulated by approximately 1.5- and 1.75-fold, respectively. Moreover, the expression level of lva-miR-D9-3p and lva-miR-4850 had the same pattern, where alteration of expression was not observed at 6 hpi but was down-regulated by approximately two-fold at 24 hpi. The upregulated miRNAs were lva-miR-2169-3p and lva-miR-7170-5p, where the expression level was increased approximately two- and three-fold, respectively, at 6 hpi, and by 2.5- and 10-fold, respectively, at 24 hpi. Changes in the expression level of lva-miR-4901 and lva-miR-92a-3p were observed at 6 hpi. The lva-miR-4901 was down-regulated by 1.7-fold at 6 hpi and returned to basal level at 24 hpi; whereas, that of lva-miR-92a-3p was increased by approximately two-fold at 6 hpi and remained unchanged at 24 hpi. The expression level of lva-miR-92b-5p was unchanged at 6 hpi but increased by approximately 1.75-fold at 24 hpi.

### Prediction of miRNA targets

**Figure 2:**
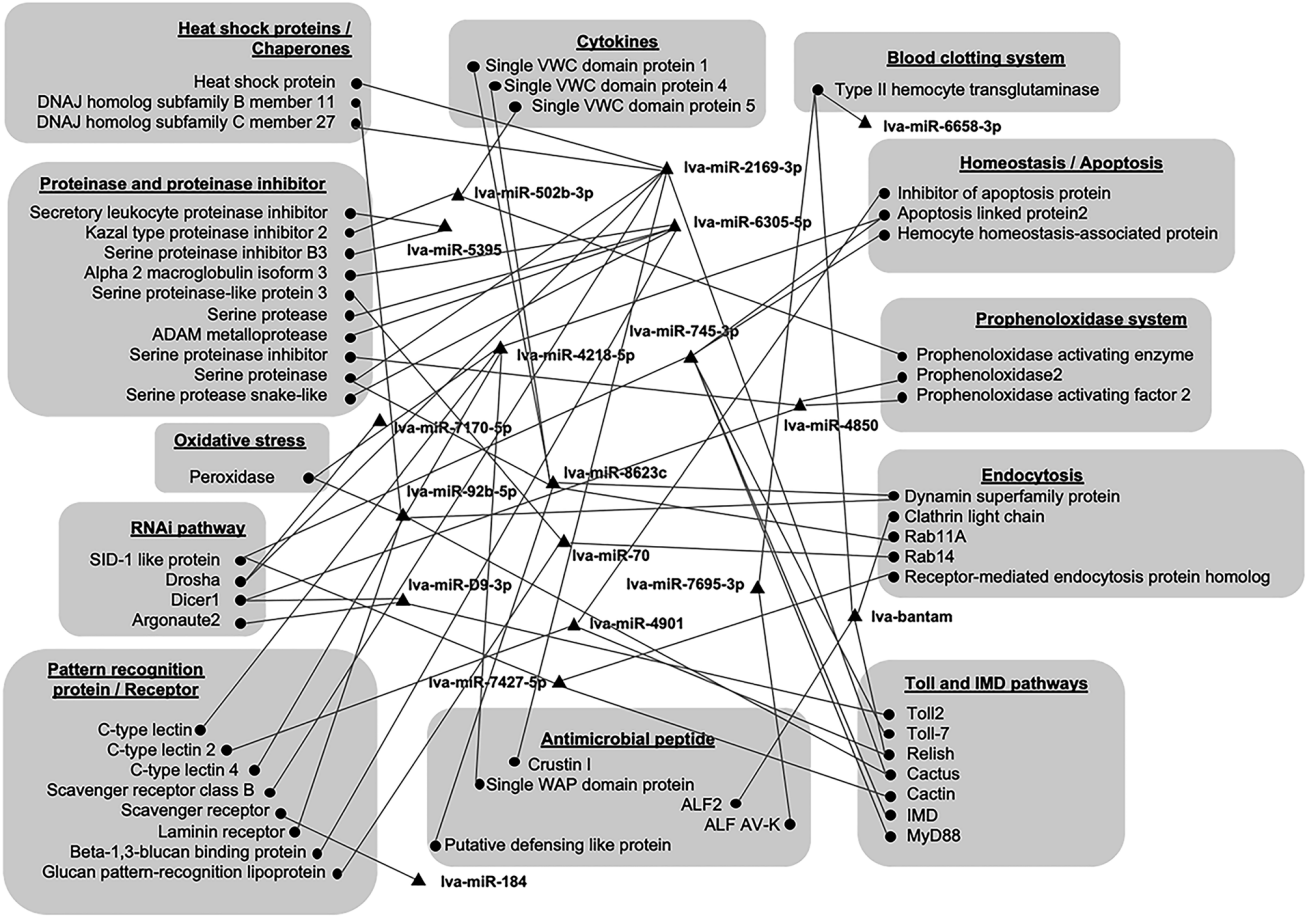
Predicted interactions between miRNAs and target *P. vannamei* immune genes. The target genes of VP_AHPND_-responsive miRNAs were predicted against the *P. vannamei* transcriptome database using the developed miRNA target prediction program. Target genes were grouped according to immune-related function.

The function of miRNA on gene expression regulation depends on its ability to directly bind to the target mRNA. Therefore, identification of the target mRNA could provide clues regarding the role of miRNA in the shrimp’s immune response against VP_AHPND_ infection. The transcriptome database of VP_AHPND_-infected *P. vannamei*^22^ was used for mRNA target prediction using CU-Mir (https://cumir.shrimp-irn.org/), an in-house developed miRNA target prediction program. Although several target genes were predicted, this study emphasized the immune-related genes by targeting VP_AHPND_-responsive miRNAs. Several shrimp immune-related genes in the groups of heat shock proteins/chaperones, cytokines, blood clotting system, proteinase and proteinase inhibitors, homeostasis/apoptosis, proPO system, oxidative stress, RNAi pathway, antimicrobial peptide, pattern recognition protein/receptor, Toll and immune deficient (IMD) pathways, and endocytosis were predicted to be their targets (Fig. 2).

### Expression analysis of the target mRNA of lva-miR4850 in *P. vannamei* shrimps by qRT-PCR

**Figure 3:**
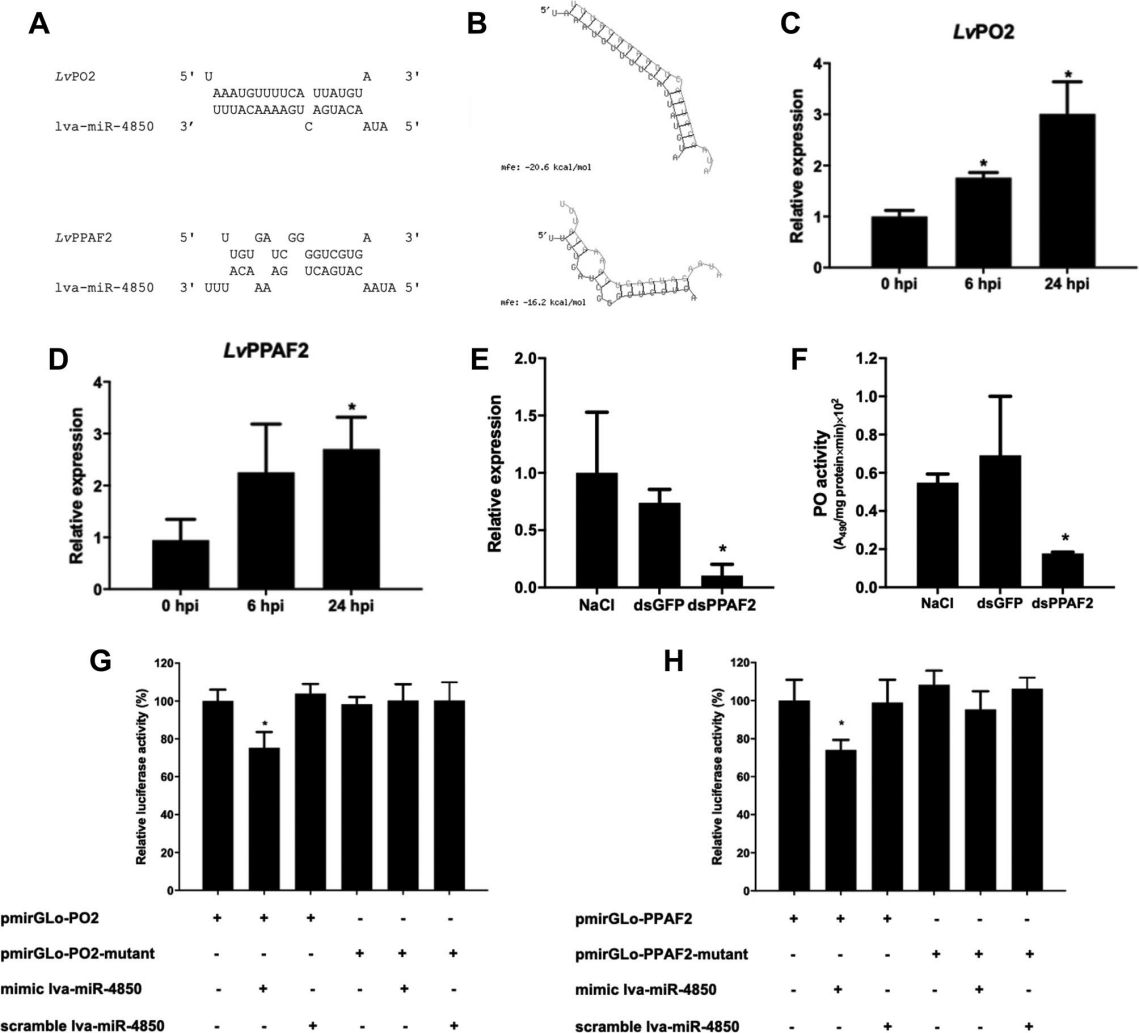
The lva-miR-4850 target identification. (**A**,**B**) Binding of lva-miR-4850 to target mRNA, as predicted using the (**A**) in-house and (**B**) RNAhybrid software. (**C**,**D**) Relative expression levels of (**C**) *PO2* and (**D**) The expression of *PPAF2*, the mRNA targets of lva-miR-4850, in response to VP_AHPND_ infection in *P. vannamei* hemocytes. Relative expression levels were determined by qRT-PCR and standardized against *EF-1α* as the internal control. (**E**) The efficiency of the *PPAF2* knock-down, shown as the *PPAF2* expression level, in shrimp hemolymph at 48 h post dsRNA injection relative to *EF-1α* expression as an internal control. (**F**) The PO activity of shrimp hemolymph was determined in the *PPAF2* knock-down shrimp (dsPPAF2) compared with the control groups (NaCl and GFP-dsRNA injection). (**G**,**H**) Synthetic mimic lva-miR-4850 or scramble lva-miR-4850 were co-transfected with the pmirGLO-target or pmirGLO-target-mutant into HEK293-T cells in order to confirm the interaction of lva-miR-4850 with (**G**) *PO2* and (**H**) *PPAF2*. Luciferase activity was measured at 48 h after transfection. Data are shown as the mean ± 1 SD, derived from three independent repeats. Asterisks indicate significant differences at *P* < 0.05 (DMRT).

Based on the miRNA-target prediction, the VP_AHPND_-responsive miRNA lva-miR4850 was selected for further miRNA/target interaction analysis. Notably, lva-miR4-850 was predicted to target the 3′-untranslated (3ʹ-UTR) of the *PO2* gene and the open reading frame (ORF) of the *PPAF2* gene of the proPO system (Fig. 3A,B). The expression analysis of putative lva-miR4850 target genes revealed that the *PO2* and *PPAF2* genes were up-regulated after VP_AHPND_ infection in *P. vannamei* hemocytes by approximately 1.8- to 3-fold (Fig. 3C,D), and these were negatively correlated to that of lva-miR-4850 (Fig. 1C). The results suggested that *PO2* and *PPAF2* might be lva-miR-4850 target genes.

### The effect of *PPAF2* silencing on PO activity in shrimps

Because the involvement of *PPAF2* in the proPO activating cascade has not been reported in *P. vannamei*, RNA interference (RNAi) was used to evaluate that speculation. First, the efficiency of the *PPAF2* knock-down in *P. vannamei* was confirmed by RT-qPCR at 48 h post-injection with PPAF2-dsRNA (Fig. 3E). The PO activity in the hemolymph of *PPAF2*-knocked down shrimps at 48 h post-injection with PPAF2-dsRNA was declined compared to that in the control groups injected with either GFP-dsRNA or NaCl (Fig. 3F). This implied that *PPAF2* plays a role in the PO cascade of *P. vannamei* immunity.

### Confirmation of target mRNA of lva-miR-4850 by dual-luciferase reporter assay

To confirm the miRNA/target interaction, two pmirGLO vectors, pmirGLO-PO2 and pmirGLO-PPAF2, containing DNA fragments corresponding to the putative miRNA-binding region of the *PO2* and *PPAF2*, respectively, were constructed, Also, the mutated seed region constructs, pmiRGLO-PO2-mutant and pmiRGLO-PPAF2-mutant, were prepared. All reporter plasmids were then co-transfected into HEK293-T cells with either a lva-miR-4850 mimic or a lva-miR-4850 scramble. In the presence of the lva-miR-4850 mimic, the luciferase activity observed from cells transfected with pmirGLO-PO2 (Fig. 3G) and pmirGLO-PPAF2 (Fig. 3H) were reduced by around 25% compared to that with the corresponding control mutant construct. The reduction in firefly luciferase expression indicates the binding of lva-miR-4850 to the cloned miRNA target sequence. On the other hand, the mutated seed sequence of lva-miR-4850 did not affect luciferase activity compared to that of the control group. These results indicated that the *PO2* and *PPAF2* were specific target genes of lva-miR-4850.

### Suppression of *PO2 *and *PPAF2* by lva-miR-4850 in VP_AHPND_-infected *P. vannamei* diminishes PO activity

**Figure 4:**
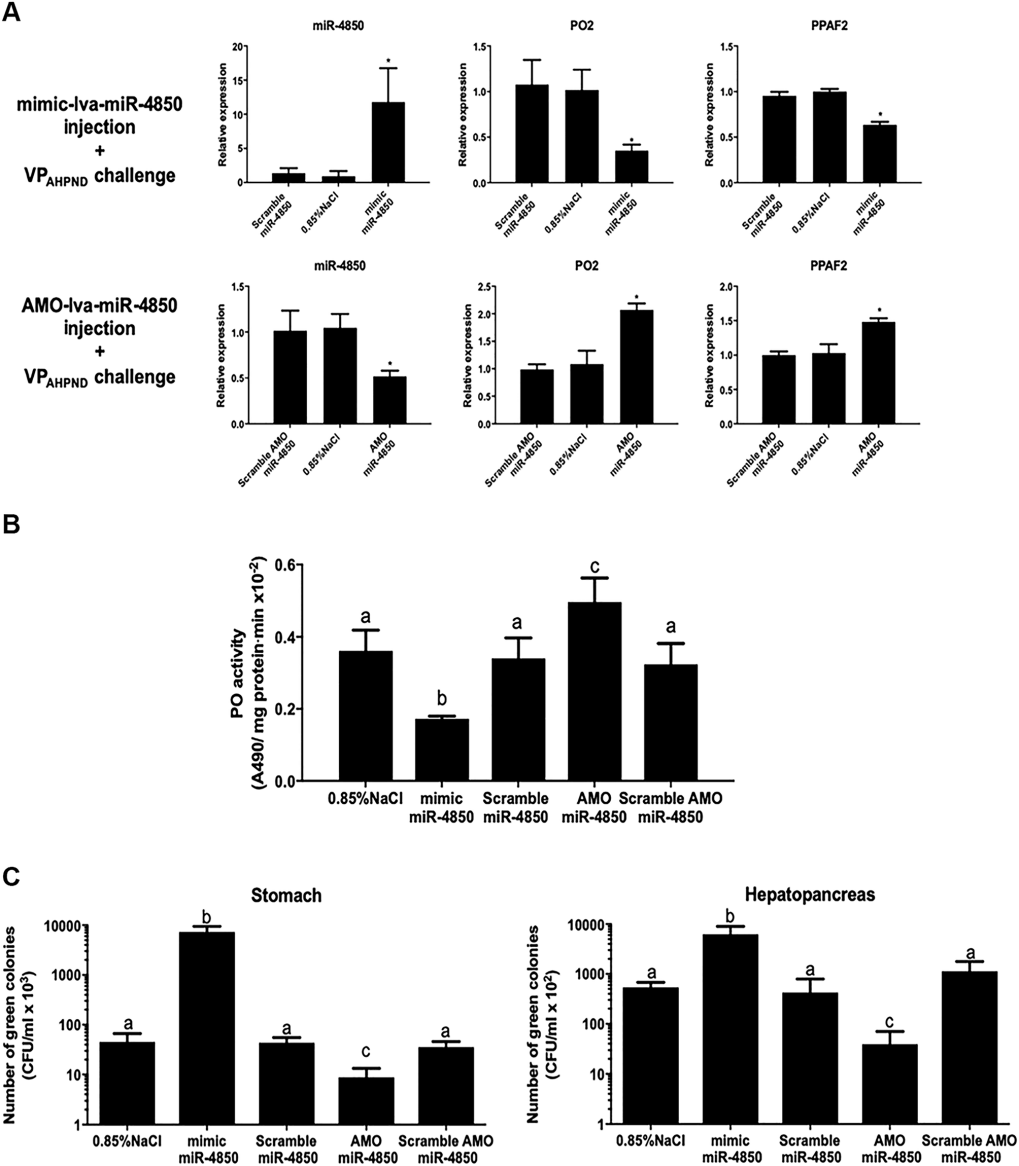
Regulation of *PO2* and *PPAF2* expression by lva-miR-4850 in VP_AHPND_-infected shrimp. (**A**) Expression analysis of lva-miR-4850, *PO2*, and *PPAF2* and (**B**) hemolymph PO activity (24 hpi) in mimic-lva-miR-4850-, scramble mimic-lva-miR-4850-, AMO-lva-miR-4850-, scramble AMO-lva-miR-4850-, or 0.85% (w/v) NaCl-injected shrimps challenged with VP_AHPND_. (**C**) Shrimps were injected as above, 24 h later infected with 1 × 10^6^ CFU/mL VP_AHPND_ by immersion, and then at 24 hpi the stomach and HP were harvested to determine the number of VP_AHPND_ by dotting on TCBS agar and subsequently counting the total number of viable green colonies (CFUs). Data are shown as the mean ± 1 SD, derived from three independent repeats. Lowercase letters and asterisks indicate significant differences at *P* < 0.05 (DMRT).

To investigate whether lva-miR-4850 regulates *PO2* and *PPAF2* in *P. vannamei*, in vivo RNAi experiments were performed. Shrimps were injected with either mimic-lva-miR-4850, scramble mimic-lva-miR-4850, AMO-lva-miR-4850, scramble AMO-lva-miR-4850, or 0.85% (w/v) NaCl. After 24 h post-injection, shrimps were challenged with VP_AHPND_ for 24 h and then the level of lva-miR-4850 expression and PO activity were measured. The lva-miR-4850 transcription level, as determined by qRT-PCR increased significantly (approximately tenfold) in mimic-lva-miR-4850-injected shrimps compared to that in the scramble mimic-lva-miR-4850 and 0.85% (w/v) NaCl-injected shrimps. The transcription levels of *PO2* and *PPAF2* exhibited a negative correlation to that of lva-miR-4850, as expected. The *PO2* and *PPAF2* expression levels were significantly decreased (approximately 4- and 1.5-fold, respectively) in mimic-lva-miR-4850-injected shrimps compared to the control groups (Fig. 4A). As the *PO2* and *PPAF2* expression level was decreased after injection of mimic-lva-miR-4850, the corresponding proPO activity was significantly (about twofold) decreased when compared to the control groups (Fig. 4B).

On the other hand, shrimps injected with AMO-lva-miR-4850, to inhibit lva-miR-4850, and then challenged with VP_AHPND_ exhibited a significant (approximately two-fold) reduction in the lva-miR-4850 expression level compared to the control groups. Considering the *PO2* and *PPAF2* expression levels, an inverse relationship between lva-miR-4850, *PO2*, and *PPAF2* was observed*.* The *PO2* and *PPAF2* expression levels were significantly increased (approximately 2- and 1.5-fold, respectively) in AMO-lva-miR-4850-injected shrimps as compared to the controls (Fig. 4A). In addition, shrimps injected with AMO-lva-miR-4850 and then challenged with VP_AHPND_ had an approximately 1.5-fold higher proPO activity than in the control groups (Fig. 4B).

### Effect of mimic and AMO lva-miR-4850 on the number of bacteria in the stomach and hepatopancreas (HP) of VP_AHPND_-infected *P. vannamei*

**Figure 5:**
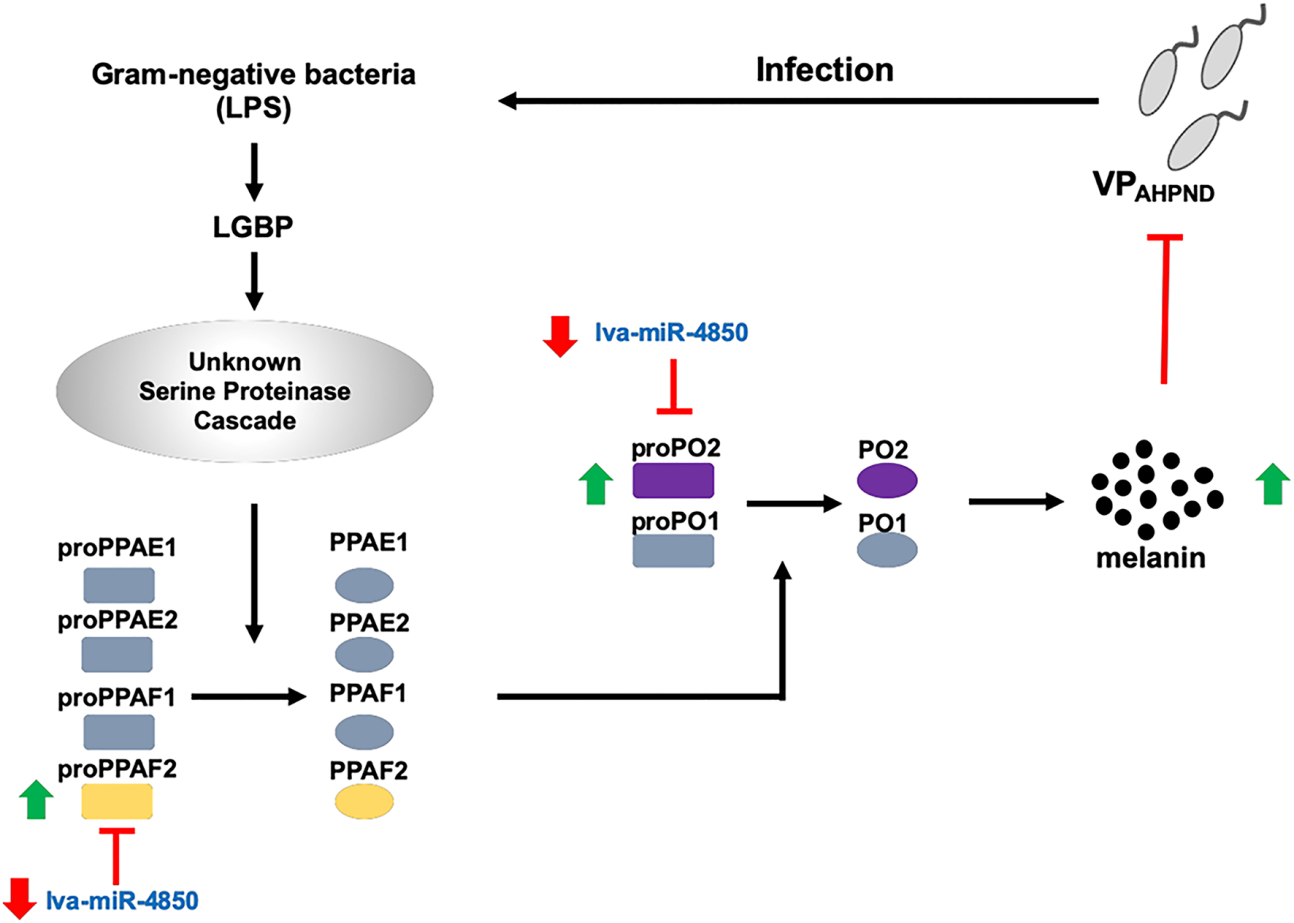
Schematic representation of how the lva-miR-4850 modulates the proPO system upon VP_AHPND_ infection.

We further investigated the effect of mimic-lva-miR-4850- and AMO lva-miR-4850-injection on the number of bacterial cells in VP_AHPND_ targeted shrimp tissues, such as the stomach and HP. The amount of total *Vibrio* sp. in the shrimp’s stomach and HP was counted at 24 hpi with VP_AHPND_, which was 48 h after injection with one of mimic-lva-miR-4850, scramble mimic-lva-miR-4850, AMO-lva-miR-4850, scramble AMO-lva-miR-4850, or 0.85% (w/v) NaCl injection. The number of green colonies (CFU/ml), representing Vibrio, in both the stomach and HP increased in shrimp injected with mimic-lva-miR-4850. The number of Vibrio in the AMO-lva-miR-4850-injected shrimps was approximately tenfold lower than in the scramble AMO-lva-miR-4850- or 0.85% (w/v) NaCl-injected groups (Fig. 4C). Taken together, we conclude that down-regulation of lva-miR-4850 upon VP_AHPND_ infection allowed the *PO2* and *PPAF2* to be expressed, resulting in proPO system activation and melanization (Fig. 5).

## Discussion

Although miRNAs have been reported to play crucial roles in regulating immune responses against virus infection in crustaceans^18^, their role in antibacterial responses has been reported previously^23^. DEMs of Vibrio-challenged animals have been widely studied. In zebrafish larvae, the miRNAs and mRNAs expression profiles were analyzed upon *V. parahaemolyticus* infection. It was found that 37 known zebrafish miRNAs were differentially expressed in the infection group. Among them, dre-miR-205-3p, dre-miR-141-5p, dre-miR-200a-5p, dre-miR-92a-2-5p, dre-miR-192, and dre-miR-1788 may play important roles in the innate immune response by regulating target immune genes^24^. In *Scylla paramamosain*, 161 miRNAs were found to be significantly differentially expressed during *V. parahaemolyticus* challenge and their potential targets were immune-related genes^25^. In *P. monodon,* miR-4286 and miR-107b were significantly changed after VP_AHPND_ infection which might regulate *dystrophin* expression, calcium concentration upon infection^26^. In *P. vannamei*, a total of 83 miRNAs were significantly differentially expressed in hemocytes upon VP_AHPND_ infection^27^. However, there are still mysteries about the roles of miRNAs in regulating the immune response during VP_AHPND_ infection.

In this study, we analyzed expression of VP_AHPND_-responsive miRNAs in *P. vannamei* hemocytes to better understand the function of miRNAs in shrimp antibacterial immunity using sRNA-Seq. In our study, 20 miRNAs were identified as a differentially expressed miRNA homologs in shrimp hemocytes upon VP_AHPND_ infection, and they were identified by searching for homologs against the vertebrate and invertebrate miRNAs, whereas the miRNA homologs from Zheng et al. (2018)^27^ were identified based only on the data of invertebrate species. Comparing these two sRNA-Seq data sets, only lva-miR-71 shared a similar expression profile. In our study, four out of 10 miRNAs (lva-miR-9000, lva-miR-7170-5p, lva-miR-92a-3p, and lva-miR-2169-3p) were confirmed to be significantly differentially expressed in shrimp hemocytes upon VP_AHPND_ infection at 6 hpi, as identified from the sRNA-Seq data. It is known that high-throughput sequencing tends to generate false-negative results; therefore, qRT-PCR is generally used to confirm the expression of transcript^28–30^. The expression levels of 10 miRNAs were differentially expressed after VP_AHPND_ challenge.

In combination with the previous transcriptomic data of VP_AHPND_-infected *P. vannamei* hemocytes^22^, we analyzed the data based on the negative correlation in gene and miRNA expression and the complementary miRNA/mRNA target prediction to further define the miRNA/mRNA interaction involved in antibacterial responses. According to Zheng et al. (2018)^27^, 12 miRNAs and their predicted target genes are possibly involved in modulating several immune-related processes in the pathogenesis of AHPND. In our study, we found that VP_AHPND_-responsive miRNAs might regulate several shrimp immune genes involved in heat shock proteins/chaperones, cytokines, blood clotting system, proteinase and proteinase inhibitors, homeostasis/apoptosis, proPO system, oxidative stress, RNAi pathway, antimicrobial peptide, pattern recognition protein/receptor, Toll and IMD pathways, and endocytosis (Fig. 2).

Based on the miRNA target function, we were interested in the down-regulated miRNAs in VP_AHPND_-infected *P. vannamei*, especially lva-miR-4850, whose target genes (*PO2* and *PPAF2*) are major components of the proPO system. Upon bacterial infection, the shrimp proPO system is crucial in shrimp antibacterial immunity. It produces melanin and cytotoxic intermediates for bacterial sequestration^31^. The proPO is a key enzyme in the melanization cascade that also participates in cuticle sclerotization, wound healing, and pathogen killing^32^.

The PPAFs, also known as proPO‐activating enzymes, are the terminal components of the proPO activation that directly convert proPO into PO, which then catalyzes the oxidation of phenolic compounds to form melanin^33^. The PPAFs are members of the CLIP subfamily, specific serine proteases that are characterized by the presence of one or more disulfide bond patterns named clip domains in the N‐terminus^34,35^. In the shrimp *P. monodon*, *PmPPAF* was up-regulated in response to WSSV infection and played an important role in the activation of the PO system^36^. Although *PPAF2* has not been characterized in *P. vannamei*, its expression level was found to be significantly increased after VP_AHPND_ infection in *P. vannamei* hemocytes, while *PPAF2* suppression by RNAi decreased the PO activity. This suggested that the *PPAF2* of *P. vannamei* is involved in the pro-PO activation pathway.

The functions of miRNAs in regulating the proPO system have been reported in shrimps. Ten miRNAs (let-7, miR-184, miR-1, miR-275, miR- 9a, miR-279, miR-965, miR-71*, miR-71, and miR-8*) were found to be up-regulated when the hemocytic PO activity was inhibited, whereas they were down-regulated when the PO activity was activated. These results suggest that these miRNAs played important roles in the negative regulation of the proPO system^37^.

The pmo-miR-315 in *P. monodon* was reported to enhance viral replication by regulating the proPO system through the inhibition of *PmPPAE3* gene expression^38^. As expected, a negative correlation in the expression level of lva-miR-4850 and the target genes, *PO2* and *PPAF2*, was observed in this study in *P. vannamei* hemocytes after VP_AHPND_ infection, while a decreased bacterial number in the HP and stomach of VP_AHPND_-infected shrimps after AMO-lva-miR-4850 injection was observed. On the other hand, following VP_AHPND_ infection the bacterial number in mimic-lva-miR-4850 challenged *P. vannamei* was higher than that in those challenged with exogenous lva-miR-4850. These indicated that lva-miR-4850 plays a crucial role in modulating the two key genes of the proPO system. In conclusion, lva-miR-4850 was down regulated in shrimp hemocytes upon VP_AHPND_ infection, which allows *PO2* and *PPAF2* to be expressed and so activation of the proPO system.

## Methods

### Shrimp samples

Healthy shrimps, weighing 2–4 g, were obtained from a commercial farm at Petchaburi province (Thailand) and acclimatized in rearing tanks at ambient temperature (30 ± 2 °C), water salinity of 20 parts per thousand, and constant aeration before use in the experiments.

### Mimic, scramble mimic, anti-miRNA oligonucleotide (AMO), and AMO scramble RNA

The mimic, scramble mimic, AMO, and AMO scramble RNA of lva-miR-4850 used for in vitro and in vivo experiments were synthesized by the Shanghai GenePharma Co., Ltd., P.R. China (Table S2).

### Bacterial challenge experiments

The VP_AHPND_ inoculum was prepared by culturing the bacteria overnight in 3 mL of tryptic soy broth (TSB) containing 1.5% (w/v) sodium chloride (NaCl) at 30 °C with shaking at 250 rpm. The starter culture was then transferred to 200 mL TSB with 1.5% (w/v) NaCl and further incubated at 30 °C and 250 rpm until the optical density at 600 nm (OD_600_) reached 2.0 (approximately 10^8^ colony forming units [CFU]/mL). Each shrimp was then challenged with VP_AHPND_ by immersion in the bacterial inoculum at a final concentration of 1.5 × 10^6^ CFU/mL (LD_50_ = 24 h).

### RNA extraction

The hemolymph (500 µL) of VP_AHPND_-challenged shrimps at 0 and 6 h post-infection (hpi) was drawn out from the ventral sinus using a sterile syringe pre-loaded with an equal volume of anticoagulant (27 mM sodium citrate, 336 mM sodium chloride, 115 mM glucose, and 9 mM EDTA, pH 5.6)^39^. Hemocytes were then collected by centrifugation at 800×*g* for 10 min at 4 °C. The hemocytes from 30 individuals were pooled and extracted for total small (s)RNA using a mirVana miRNA Isolation Kit (Ambion, Life Technologies) following the manufacturer's protocol. The total sRNA quality was evaluated on Agilent 2100 Bioanalyzer using a Small RNA Kit (Agilent). The RNA concentrations were determined by Qubit RNA HS Assay Kit on the Qubit 2.0 flourometer (ThermoFisher Scientific).

### sRNA-Seq and data analysis

The cDNA libraries of sRNA from VP_AHPND_-infected shrimp hemocytes at 0 and 6 hpi were constructed using the TruSeq Small RNA Library Preparation Kit (Illumina) according to the manufacturer's instruction. The indexed libraries were normalized, pooled, and then, sequenced with a PhiX control spiked at 7.5% using MiSeq Reagent Kits v2 (Illumina) in a MiSeq sequencer (Illumina). The sRNA-Seq analysis was performed as previously described by Boonchuen et al. (2020)^22^. Briefly, the Galaxy instance (https://usegalaxy.org/) was used for 5′- and 3′-adapter trimming and for quality control of raw reads^40^. The high-quality sRNA sequences of a length shorter than 18 nucleotides and longer than 24 nucleotides were removed. The contaminating RNA, such as mRNA, rRNA, and tRNA, was also removed. The remaining sequences were then searched against miRBase 22.1 (http://www.mirbase.org/) to identify known miRNA homologs. Differentially expressed miRNA (DEM) analysis was performed as follows. Firstly, the read no. of each miRNA from the treatment and control groups were normalized to the total no. of reads of that respective library at the same orders of magnitude. Formula:

$$\text{Normalized expression level}=\frac{\text{miRNA expression level}}{\text{total expression level of the sample}}\text{normalized magnitude}$$

Secondly, the normalized expression level was used to calculate the fold change and *P*-value^41^. Finally, we used the *P*-value method to ensure the statistical significance of DEMs, which of interest were generated using *P*-value *P* < 0.05 and log_2_Ratio ≥  ± 1.

### Quantitative (q)RT-PCR analysis

The miRNAs of interest, such as lva-miR-9000, lva-miR-D9-3p, lva-miR-4850, lva-miR-2169-3p, lva-miR-7170-5p, lva-miR-4901, lva-miR-92a-3p, lva-miR-92b-5p, lva-miR-184, and lva-miR-8522c, were selected for expression analysis using stem-loop qRT-PCR. Pooled total sRNA from VP_AHPND_-infected shrimp hemocytes at 0, 6, and 24 hpi was prepared using the mirVana miRNA Isolation Kit (Ambion, Life technologies). The total sRNA as a template, the stem-loop RT primers specific to each miRNA, and the internal control U6 (Table S3) were used to synthesize the first-strand cDNA using the RevertAid First-strand cDNA Synthesis Kit (Thermo Fisher Scientific). Expression of the U6 gene was used as the internal control. Stem-loop qRT-PCR was performed using an appropriate amount of cDNA for each gene, specific oligonucleotide forward primer (Table S3), and QPCR Green Master Mix (Biotechrabbit) in the MiniOpticon™ Real-time PCR System (Bio-Rad). Thermal cycling was performed under the following conditions: 95 °C for 3 min followed by 40 cycles of 95 °C for 30 s, 60 °C for 30 s, and 72 °C for 30 s. The relative expression level compared to that of U6 was calculated.

### Prediction of miRNA targets

The miRNA targets were identified by searching for the miRNA complementary sequence in the transcriptome data of VP_AHPND_-challenged *P. vannamei* hemocytes^22^ using the CU-Mir software (http://shrimp-irn.org/mirtarget/index.php) that was developed in-house^42^. The CU-Mir software was used to search for locations on mRNA targets that seed sequences (2–8 nucleotides from the 5′ end) of miRNA that can bind perfect complementary or one mismatch at any different region; an open reading frame (ORF); 3′-untranslated region (UTR) and 5′-UTR. The percent complementary between sequences was calculated from the number of nucleotides that complementarily bind to the target mRNAs per total length of the miRNA sequence. The overall complementarity of miRNA to the target mRNA cut off was set at 40%. RNAhybrid software (http://bibiserv.techfak.uni-bielefeld.de/rnahybrid/) was also used to predict genes targeted by miRNAs using a free energy of <  − 15.0 kcal/mol^42^.

### Dual-luciferase reporter assay

The luciferase reporter system was used to confirm the interaction between the miRNA of interest, lva-miR-4850, and the target *PO2* and *PPAF2* genes. The gene fragments containing the predicted lva-miR-4850 target sites, such as the 3′UTR of the *PO2* gene and the ORF of the *PPAF2* gene, were amplified from the cDNA of the VP_AHPND_-infected *P. vannamei* hemocytes using specific primers (Table S3). Those gene fragments were subsequently cloned into the pmiRGLO plasmid (Promega) at the 3′UTR of firefly luciferase and pmiRGLO-stop-mutant to produce the pmirGLO-PO2 and pmirGLO-PPAF2 plasmids, respectively.

To construct the experimental control, the binding element at nucleotide positions 2–8 was mutated by QuickChange II XL Site-Directed Mutagenesis Kit (Agilent Technology). Briefly, the primers were designed by switching the bases of the seed sequence from purine to pyrimidine or pyrimidine to purine with a melting temperature (Tm) of ≥ 78 °C. The recombinant pmiRGLO-PO2 and pmiRGLO-PPAF2 were then amplified by *PfuUltra*-HF DNA polymerase (Agilent Technology) and the PCR product was treated with *Dpn*I restriction enzyme. The *Dpn*I-treated PCR product was further transformed into *E. coli* Top10. The mutant plasmids, pmiRGLO-PO2-mutant and pmiRGLO-PPAF2-mutant, were confirmed by sequencing (Figure S1).

For each plasmid, 200 ng were co-transfected into HEK293-T cells along with 20 pmol of mimic lva-miR-4850 or scramble lva-miR-4850 (GenePhama) using the Effectene transfection reagent (Qiagen). After 48 h post-transfection, the activity of Firefly and Renilla luciferases were measured using the Dual-Luciferase Reporter assay system (Promega) following the manufacturer's instructions.

### The silencing of PPAF2 in shrimp hemocyte

To investigate whether *PPAF2* is involved in the proPO activating system in shrimps, in vivo RNAi experiments were performed. The *PPAF2* was amplified from the cDNA of *P. vannamei* hemocytes using a specific primer pair (Table S3), cloned into the pGEM-T easy vector (Promega), and used as a template for the preparation of the dsRNA specific to the *PPAF2* (dsRNA-PPAF2). In addition, the dsRNA of the *green fluorescent protein* (*GFP*; dsRNA-GFP), the negative control, was prepared from the pEGFP-1 vector (Clontech) as a template. The dsRNA-PPAF2 and dsRNA-GFP were prepared using T7 RiboMAX Express Large-Scale RNA Production System (Promega) according to the manufacturer’s instruction. The primers used for dsRNA-PPAF2 (knPPAF2-T7-F, knPPAF2-R, knPPAF2-F, and knPPAF2-T7-R) and dsRNA-GFP (knGFP-F, knGFP-R, knGFP-T7-F, and knGFP-T7-R) production are listed in Table S3. The quantity and quality of dsRNA were verified by nanodrop spectrophotometry and agarose gel electrophoresis, respectively.

*P. vannamei* of approximately 3 g body weight were divided into three groups of three individuals each. The first group (control) was injected with 150 mM NaCl, the second group (dsRNA control) with 5 μg/g shrimp of dsRNA-GFP, whilst the third group, the *PPAF2* knockdown, was injected with 5 μg/g shrimp of dsRNA-PPAF2. The hemolymph of individual shrimp was collected at 48 h post-injection. The total RNA from shrimp hemolymph was extracted by Genezol reagent (Geneaid). Subsequently, the first strand cDNA synthesis was performed. Suppression of *PPAF2* expression was determined by qRT-PCR using specific primers. The *elongation factor-1α* (*EF-1α*) was used as an internal control.

### In vivo effect of lva-miR-4850 introducing and gene silencing

Shrimps (2–3 g) were divided into five groups of three individuals each. Experimental groups were intramuscularly injected with 50 µL of 0.85% (w/v) NaCl solution containing 2 nmol of mimic-lva-miR-4850 or AMO-lva-miR-4850, while the control groups were injected with 2 nmol of scramble mimic-lva-miR-4850, or scramble AMO-lva-miR-4850, or 50 µL of 0.85% (w/v) NaCl. At 24 h post-injection, the shrimps were challenged with VP_AHPND_ as described above and at 24 hpi their hemolymph was collected. Total RNA was extracted and used for first-strand cDNA production. The expression levels of lva-miR-4850, *PO2*, and *PPAF2* were determined by qRT-PCR, as described above.

On the other hand, the stomach and HP were separately collected from three shrimps per group, homogenized, and serially tenfold diluted in sterile 0.85% (w/v) NaCl. The diluted (10^1^- to 10^6^-fold) samples were plated onto thiosulfate-citrate-bile salts-sucrose (TCBS) agar and incubated at 30 °C for 12–14 h. The bacterial colonies were then counted and calculated as CFU/mL.

### Determination of the PO activity

The PO activity was determined in the shrimp hemolymph collected at 24 hpi with miRNA-VP_AHPND_ challenge (48 h post-injection with dsRNA). The PO activity was measured using a modification to the reported method^43^. In brief, 50 μL of hemolymph was mixed with 25 μL of 3 mg/mL freshly prepared L-3, 4-dihydroxyphenylalanine (L-DOPA; Fluka), and 25 μL of 20 mM Tris–HCl (pH 8.0). The absorbance at 490 nm (A_490_) was monitored after 60 min of incubation. The amount of hemolymph proteins was measured using the Bradford method^44^. The PO activity was recorded as A_490_/mg total protein/min.

### Statistical analysis

Differences in the dual-luciferase reporter assay were analyzed using the GraphPad Prism 8.0 software with the statistical analyses, including a paired-samples *t *test, while miRNA and gene expression levels were analyzed using a paired-samples *t *test. All other numerical data was analyzed using one-way ANOVA followed by Duncan's new multiple range test (DMRT), and are presented as the mean ± one standard deviation (SD). Statistical significance was accepted at the *P* < 0.05 level.

### Ethics statement

Experiments involving animals were performed in compliance with the animal use protocol number 1823006 approved by the Chulalongkorn University Animal Care and Use Committee (CU-ACUC). The biosecurity concerns of experiments performed was reviewed and approved by the Institutional Biosafety Committee and Chulalongkorn University (CU-IBC; approval number: SCI-01-001).

## Supplementary Information

Supplementary Information.
