# Supplementary material for: Regulation of shrimp prophenoloxidase activating system by lva-miR-4850 during bacterial infection
Source: Sci Rep. 2021 Feb 15;11:3821. doi: 10.1038/s41598-021-82881-2 (PMC7884684; doi:10.1038/s41598-021-82881-2)
Supplement: Supplementary file 1 — Supplementary Information. [file 41598_2021_82881_MOESM1_ESM.pdf]

Regulation of shrimp prophenoloxidase activating system by lva-miR-4850 during bacterial infection

Pakpoom Boonchuen[1], Phattarunda Jaree[2], Kulwadee Somboonviwat[3], Kunlaya Somboonwiwat[1,4,*]

[1]Center of Excellence for Molecular Biology and Genomics of Shrimp, Department of Biochemistry, Faculty of Science, Chulalongkorn University, Thailand

[2]Institute of Molecular Biosciences, Mahidol University, Salaya, Nakhon Pathom Thailand

[3]Faculty of Engineering at Sriracha, Kasetsart University Sriracha Campus, Sriracha, Chonburi, Thailand

[4]Omics Science and Bioinformatics Center, Faculty of Science, Chulalongkorn University, Thailand

[*]Corresponding author:

Tel.: +66-2-218-5438 Fax: +66-2-218-5418

E-mail: kunlaya.s@chula.ac.th (Kunlaya Somboonwiwat)

Running title: lva-miR-4850 regulates the proPO system

Table S1 Summary of sequences identified from the sRNA libraries of *P. vannamei* hemocytes challenged with VP_{AHPND} at 0 and 6 hpi

	Number of reads	
	0 hpi	6 hpi
Raw reads	931,638	1,252,728
Passed-filter reads	817,019	1,104,193
Trimmed 3' and 5' adapter	31, 806	52,321
Size selection	30,117	49,815
Contaminating RNA	7,519	12,592
Mature microRNA homolog	22,598	37,223
pre-miRNA homolog	1,091	1,396
Unknown sRNA	103	156

Table S2 Mimic, scramble mimic, anti-miRNA oligonucleotide (AMO), and AMO scramble RNA sequences

Gene	Name	Sequence	
		Sense（5'-3'）	Antisense（5'-3'）
lva-miR-4850	mimic	AUAACAUGACUGAAAACAUUU	AUGUUUUCAGUCAUGUUAUUU
	Scramble mimic	GUUAAUACCUAAGAUAUACAA	GUAUAUCUUAGGUAUUAACUU
	AMO	AAAUGUUUUCAGUCAUGUUAU	
	Scramble AMO	GAUUUAUCCAUAUUGAUUAGU	

Table S3 Primers used in this study

miRNA/Gene name	Primer name	Sequence (5'-3')	
lva-miR-4850	miR-4850-F	GTTGGGGATAACATGACTGAAA	Expression analysis
	miR-4850_stem loop RT	GTTGGCTCTGGTGCAGGGTCCGAGGTATTCGCACCAGAGCCAACAAATGT	
lva-miR-8522c	miR8552c-F	TTGTGGGGCCGTGATCGT	
	miR-8552c_ stem loop RT	GTTGGCTCTGGTGCAGGGTCCGAGGTATTCGCACCAGAGCCAACCACTAT	
lva-miR-184	miR-184-F	GTTGTGGACGGAGAACTGA	
	miR-184_ stem loop RT	GTTGGCTCTGGTGCAGGGTCCGAGGTATTCGCACCAGAGCCAACCCCTTA	
lva-miR-4901	miR-4901-F	GTTGGGGGTAACTTATTTTTGG	
	miR-4901_ stem loop RT	GTTGGCTCTGGTGCAGGGTCCGAGGTATTCGCACCAGAGCCAACGTTTGT	
lva-miR-92a-3p	miR-92a-3p-F	GTTTTCGTCTCGTGTCTCG	
	miR-92a-3p_ stem loop RT	GTTGGCTCTGGTGCAGGGTCCGAGGTATTCGCACCAGAGCCAACCTAAGG	
lva-miR-7170-5p	miR-7170-5p-F	GTTGAACTGGAGGACCGAA	
	miR-7170-5p_ stem loop RT	GTTGGCTCTGGTGCAGGGTCCGAGGTATTCGCACCAGAGCCAACAGTCGG	
lva-miR-D9-3p	miR-D9-3p	GTTTCCAGAATGTTCCACT	
	miR-D9-3p_ stem loop RT	GTTGGCTCTGGTGCAGGGTCCGAGGTATTCGCACCAGAGCCAACAGTGGA	
lva-miR-92b-5p	miR-92b-5p-F	TTGTGGGGACGAGAAGCG	
	miR-92b-5p_ stem loop RT	GTTGGCTCTGGTGCAGGGTCCGAGGTATTCGCACCAGAGCCAACAAGCAC	
lva-miR-2169-3p	miR-2169-3p-F	GTTTGATTTAAAGTGGTACGCG	
	miR-2169-3p_ stem loop RT	GTTGGCTCTGGTGCAGGGTCCGAGGTATTCGCACCAGAGCCAACCCAGCT	
lva-miR-9000	miR-9000	TTTGAAGCCCCAGTGGCGCA	
	miR-9000_ stem loop RT	GTTGGCTCTGGTGCAGGGTCCGAGGTATTCGCACCAGAGCCAACTGCGCCA	
Universal primer		GTGCAGGGTCCGAGGT	
U6	U6-qRTF	GTACTTGCTTCGGCAGTACATATAC	
	U6-qRTR	TGGAACGCTTCACGATTTTGC	
PO2	LvPPO2-F	CCGTGAACAACTCCGGAAGA	
	LvPPO2-R	CTGAGATTCGAGTCGGCCTC	
PPAF2	PPAF2-RT-F	GATCCGGATATGGGGCTGTG	
	PPAF2-RT-R	CTCACGACCCCGATCACAAA	
EF-1α	EF-1α-F	CGCAAGAGCGACAACTATGA	
	EF-1α-R	TGGCTTCAGGATACCAGTCT	
PO2	Nhel-PO2-F	CAGGCTAGCGCGGTTTCTGTATTAGAGTTAG	pmiRGLO cloning
	Xbal-PO2-R	CAGTCTAGATTATTTAAACGCATTTCTTGTG	
PPAF2	Nhel-PPAF2-F	CAGGCTAGCGCGGCCCTATGGTCATCAGCAAC	
	Xbal-PPAF2-R	CAGTCTAGACACCTTCCTTGCCTGACCTTCC	
PO2	Mutate-pmiRGLO-PO2-F	GCAGATACAGAAATACTAAATGTTTTCAAATACAATAAAATGTGAAAACTGTGTTCCCTGT	Site directed mutagenesis
	Mutate-pmiRGLO-PO2-R	ACAGGGAACACAGTTTTCACATTTTATTGTATTTGAAAACATTTAGTATTTCTGTATCTGC	
PPAF2	Mutate-pmiRGLO-PPAF2-F	GGACCCGAAGATTTGTGATCGGCCAGCACAGTCTCGGTCCTGCTGTCTGCGGCGCCC	
	Mutate-pmiRGLO-PPAF2-R	GGGCGCCGCAGACAGCAGGACCGAGACTGTGCTGGCCGATCACAAATCTTCGGGTCC	
pmirGLO-mutant	Mutate-pmiRGLO-F	AGAAGGGCGGCAAGATCGCCGTGCCTAATTCTAGTTGTTTAAACGAGCTC	
	Mutate-pmiRGLO-R	GAGCTCGTTTAAACAACTAGAAATTAGGCACGGCGATCTTGCCGCCCTTCT	
PPAF2	knPPAF2-F	TTTGTGATCGGGGTCGTGAG	Cloning
	knPPAF2-R	CTTCTCCTTCTTTTCCTCCCTCC	
PPAF2	knPPAF2-F	TTTGTGATCGGGGTCGTGAG	Knock-down
	knPPAF2-R	CTTCTCCTTCTTTTCCTCCCTCC	
	T7-knPPAF2-F	TAATACGACTCACTATAGGGTTTGTGATCGGGGTCGTGAG	
	T7-knPPAF2-R	TAATACGACTCACTATAGGGCTTCTCCTTCTTTTCCTCCCTCC	

A

```
PPAF2-WT          GCGGCCCTATGGTCATCAGCAACAACGGCAGGACCCGAAGATTTGTGATCGGGGTCGTGA      60
PPAF2-mutant      GCGGCCCTATGGTCATCAGCAACAACGGCAGGACCCGAAGATTTGTGATCGGccagcacA      60
                  ****************************************************       *

PPAF2-WT          GTCTCGGTCCTGCTGTCTGCGGCGCCCCCGAGAGCTCCGGAATCTACACCAGCGTTATCT      120
PPAF2-mutant      GTCTCGGTCCTGCTGTCTGCGGCGCCCCCGAGAGCTCCGGAATCTACACCAGCGTTATCT      120
                  ************************************************************

PPAF2-WT          ATTACATGGATTGGATCCTGAATAACCTCCGTGCTTGAGGGGAAGAAGGAAGGAAGAAGG      180
PPAF2-mutant      ATTACATGGATTGGATCCTGAATAACCTCCGTGCTTGAGGGGAAGAAGGAAGGAAGAAGG      180
                  ************************************************************

PPAF2-WT          AAAGGAAGGAAGGAGGGTGGAAAGAAGGAAAAGAAGGAGAAGGAAAGAGGCAAAGGGAAG      240
PPAF2-mutant      AAAGGAAGGAAGGAGGGTGGAAAGAAGGAAAAGAAGGAGAAGGAAAGAGGCAAAGGGAAG      240
                  ************************************************************

PPAF2-WT          GAGGTGGGAGGGAGGAAAAGAAGGAGAAGGAAGGTCAGGCAAGGAAGGTG      290
PPAF2-mutant      GAGGTGGGAGGGAGGAAAAGAAGGAGAAGGAAGGTCAGGCAAGGAAGGTG      290
                  **************************************************
```

B

```
PO2-WT            GCGGTTTCTGTATTAGAGTTAGAAATGGCTATTGTGTACATTTCTTTTGTATCTGTTTAA      60
PO2-mutant        GCGGTTTCTGTATTAGAGTTAGAAATGGCTATTGTGTACATTTCTTTTGTATCTGTTTAA      60
                  ************************************************************

PO2-WT            TATTTGGATTTTGGTACATTGTTGTATAATATATTTTGTATCTGCAGATACAGAAATACT      120
PO2-mutant        TATTTGGATTTTGGTACATTGTTGTATAATATATTTTGTATCTGCAGATACAGAAATACT      120
                  ************************************************************

PO2-WT            AAATGTTTTCATTATGTATAAAATGTGAAAACTGTGTTCCCTGTAATACTGTATTATCTG      180
PO2-mutant        AAATGTTTTCAaatacaATAAAATGTGAAAACTGTGTTCCCTGTAATACTGTATTATCTG      180
                  ***********      *******************************************

PO2-WT            TAATAACTACTTACACATATCAGTATTAGATAGTTGTATATTTTATTCATTATAATCATG      240
PO2-mutant        TAATAACTACTTACACATATCAGTATTAGATAGTTGTATATTTTATTCATTATAATCATG      240
                  ************************************************************

PO2-WT            ATATTTGAACAGACGAAATGCGTTTAAATAA      271
PO2-mutant        ATATTTGAACAGACGAAATGCGTTTAAATAA      271
                  *******************************
```

Figure S1 Alignment of the wild type and mutant seed sequence of the lva-miR-4850 binding site for the *PPAF2* (A) and *PO2* (B) genes. Yellow highlight represents the lva-miR-4850 binding sequence.
